# Patients’ Perspectives on the Usability of a Blended Approach to an Integrated Intervention for Patients With Medically Unexplained Physical Symptoms: Mixed Methods Study

**DOI:** 10.2196/19794

**Published:** 2021-09-28

**Authors:** Suze Adriana Johanna Toonders, Paula Elisabeth van Westrienen, Sophie Konings, Marianne E Nieboer, Cindy Veenhof, Martijn F Pisters

**Affiliations:** 1 Department of Health Innovation and Technology Fontys University of Applied Sciences Eindhoven Netherlands; 2 Center for Physical Therapy Research and Innovation in Primary Care Leidsche Rijn Julius Health Care Centers Utrecht Netherlands; 3 Physical Therapy Research Group Department of Rehabilitation, Physical Therapy Science and Sport Brain Center Rudolf Magnus, University Medical Center Utrecht Utrecht Netherlands; 4 Physical Therapy Sciences Program in Clinical Health Sciences University Medical Center Utrecht Utrecht Netherlands; 5 Research Group Innovation of Human Movement Care University of Applied Sciences Utrecht Utrecht Netherlands

**Keywords:** usability, medically unexplained physical symptoms, blended care

## Abstract

**Background:**

Medically unexplained physical symptoms are physical symptoms, such as pain, fatigue, and dizziness, that persist for more than a few weeks and cannot be explained after adequate medical examination. Treatment for preventing the chronicity of symptoms is recommended. A promising approach is identifying patients who are at risk and subsequently offering a blended care intervention that focuses on promoting self-management while using eHealth as a supportive tool. When these interventions match with a patient’s expectations, their effectiveness grows.

**Objective:**

This study aimed to obtain more insights into usability from the patient perspective to improve future interventions.

**Methods:**

A mixed methods design (ie, the use of qualitative and quantitative data) was used. Through semistructured interviews, in-depth insights were gained into patients’ perspectives on usability. The analysis process was continuous and iterative. Data were synthesized and categorized into different themes. The System Usability Scale, which measures the usability of a system, was used to compare participants that found usability to be low, medium, or high. This study was approved by the Medical Ethical Committee Utrecht (approval number: 17-391/C).

**Results:**

Saturation was reached after interviewing 13 participants. The following four themes emerged from the interviews: motivations and expectations prior to participating in the program, the applicability of e-coaching, the role of health care professionals, and the integrated design of the blended approach.

**Conclusions:**

The successful implementation of integrated blended care interventions based on patients’ perspectives requires matching treatments to patients’ individual situations and motivations. Furthermore, personalizing the relative frequency of face-to-face appointments and e-coaching can improve usability.

## Introduction

Medically unexplained physical symptoms (MUPS) are physical symptoms that persist for more than a few weeks and cannot be explained after adequate medical examination [1]. MUPS are a serious concern, since approximately 25% to 50% of symptoms remain unexplained in primary care [2,3]. Patients with MUPS experience symptoms such as pain, fatigue, and dizziness [4]. These symptoms often have a major impact on daily life and result in a high burden for patients with MUPS [5]. MUPS can be divided into the following three stages: mild, moderate, and chronic [6]. These stages are based on the frequency of consulting a general practitioner, the duration of symptoms, and the physical and psychological dysfunctions experienced [6]. Existing research on treatment for the chronic stages of MUPS has provided valuable insights, and recommended interventions have included cognitive behavioral therapy, exercise therapy, and neuroscience education [7]. Treatment for preventing the chronicity of symptoms has been recommended in order to reduce the severity of symptoms and the direct and indirect costs of care [8,9]. This is in line with the general trend in health care policy; policies nationwide aim to strengthen health programs to prevent diseases and address risk factors [10]. Health care is thereby changing its focus from cures and care to behavior and health [11].

In order for programs to succeed in shifting their focus to behavior and health, these programs must include proactive and indicated prevention [12]. A first step is identifying patients who are at risk for developing chronicity [13,14]. Moreover, literature has shown that programs and interventions should focus on promoting patients’ self-management [15,16]. eHealth can serve as a supportive tool for both personalization and the promotion of self-management [17,18]. eHealth is not only supportive of usual therapeutic guidance but is also a substantial element of interventions as a whole [19]. This is referred to as *blended care*—the combination of face-to-face contact with integrated web-based applications [20]—or as *e-coaching*, which is defined as “the use of technology during coaching to motivate and stimulate (groups of) people to change attitudes, behaviors, and rituals” [21,22].

When these interventions match patients’ expectations, sustainable changes in patients are achieved more effectively [23]. More insights into usability from the patient perspective can further improve these interventions [24,25]. For example, from the patient perspective, interventions should be easy to use and acceptable. This usability, which is defined as “the quality of a system with respect to ease of learning, ease of use and user satisfaction” [26], can be measured.

The objective of this study was to gain more understanding into patients’ perspectives on the usability of integrated blended care interventions. In order to do so, this study analyzed a recent proactive, multidisciplinary, and integrated blended care intervention that was developed to prevent chronicity in patients with MUPS in primary care [27,28]. At-risk patients were identified by using electronic medical records [29]. e-Coaching was used to integrate technology into the intervention. The main goals were to (1) promote self-management among patients and (2) provide patients with insights into dealing with their complaints.

## Methods

### Study Design and Setting

A mixed methods design (ie, the use of qualitative and quantitative data) was used. Through semistructured interviews, qualitative data were gathered in order to gain an in-depth understanding of usability from patients’ perspectives. System Usability Scale (SUS) scores (low, medium, and high) were compared to responses in the interviews, which allowed us to gain better insight into the relationship between identified themes from interviews and experienced usability. This study was approved by the Medical Ethical Committee of University Medical Center Utrecht (approval number: 17-391/C).

### Participants

Patients who participated in the PARASOL intervention were eligible for inclusion. To be included in the intervention, all patients (aged ≥18 years) must have had ≥5 consultations with their general practitioner in the past 12 months. Of these consultations, ≥3 had to be classified as “suggestive of MUPS” based on 1 of the 104 International Classification of Primary Care codes. Patients with medical and psychiatric diagnoses were excluded [28]. Only participants in the PARASOL intervention who provided informed consent for this follow-up study were invited. In order to obtain rich data, stratified purposeful sampling was conducted based on the outcomes of the SUS. Patients with validated SUS scores of <70, between 70 and 80, and >80 were included; these represent low, medium, and high scores for usability, respectively [30].

### Measurements

Qualitative data were collected in one-to-one semistructured interviews, which were conducted at an agreed-upon location. A second researcher was available to play the role of observer. The topic list for the interviews was based on the theoretical construct of De Bleser et al [26] and supplemented with the determinants of health care innovation that were selected and developed by the Netherlands Organization for Applied Scientific Research [31] (Textbox 1). The quantitative data consisted of the outcomes of the SUS. The SUS has high reliability [30] and contains 10 questions on the usability of a system [32]. Questions were answered on a numeric rating scale with scores that range from 1 to 5 (“strongly agree” to “strongly disagree”). The SUS was administered at the end of the intervention. The demographic data consisted of age, gender, and educational level (basic, intermediate, and high). Educational levels were derived from the Standard Classification of Education used by Statistics Netherlands [33].

**Outline of the interview guide [26]. The key areas are shown.**

**Performance**

- Impact of use environment
- Impact of user characteristics
- Ease of the manipulation of the device

**Satisfaction**

- Physical dimension
- Privacy dimension
- Human interaction
- Self-concept
- Routine
- Sustainability

**Acceptability**

- Acceptance for daily life use
- Willingness to pay for device

### Procedure

Qualitative data were collected from semistructured interviews within 4 weeks after participants completed the PARASOL intervention to avoid recall bias. Interviews took place in patients’ homes or in one of the participating health care centers, depending on the preferences of the patients. Before the interview started, procedures regarding sound recording and the coding of data were explained, after which permission was requested from the participants. Quantitative data were collected for the randomized controlled trial PARASOL (Evaluation of a Proactive Preventive Program in Patients With MUPS; trial registration number: NL57931.041.16) [28]. Demographic data were retrieved from baseline measurements. After 3 months, upon the completion of the PARASOL intervention, SUS scores were gathered.

### PARASOL Intervention

**Figure 1:**
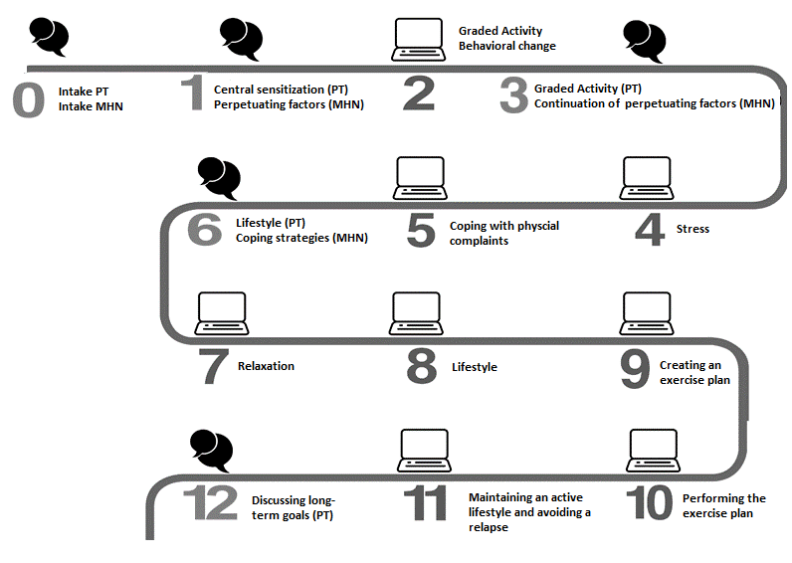
Overview of the PARASOL intervention. The text cloud images indicate face-to-face contact with a PT and MHN. The computer images indicate modules of e-coaching. MHN: mental health nurse; PT: physical therapist.

The PARASOL intervention was a 12-week integrated blended care intervention that consisted of 4 face-to-face consultations with a mental health nurse and 5 physical therapy sessions and was supplemented with e-coaching (Figure 1). e-Coaching consisted of information modules and videos on self-management and educative themes, videos and instructions on prescribed home exercises, and assignments for gradually increasing physical activity. The intervention aimed to improve patients’ perceptions of symptoms and identify modifiable risk factors of chronicity by providing therapeutic neuroscience education and promoting self-management. The intervention also aimed to promote an active lifestyle by using a cognitive behavioral approach and graded activities. Health care professionals were instructed on how to treat patients with moderate MUPS during a 2-day training session. Beyond the program itself, instructions included presentations on the study population, central sensitization, therapeutic neuroscience education, graded activities, and perpetuating factors. Furthermore, health care professionals were instructed on how to integrate e-coaching during the intervention. They were, for instance, guided on how to personalize general themes and instructed to ask patients about whether they understood information that is given on web-based platforms. All health care professionals received a guideline after finishing the training.

### Data Analysis

Interviews were recorded and transcribed verbatim, and transcriptions were checked by 2 researchers. Within 1 week after completing the interviews, a summary was sent to all participants. This member check verified whether interpretations were correct. After the initial interviews were conducted, the interviewer added other questions based on the themes that emerged from these interviews. Both researchers encoded meaningful text fragments independently, and a set of preliminary concepts and codes was generated. The analysis process was continuous and iterative. Data were synthesized and categorized into 4 different themes. In the last stage of the analysis, for each theme, interview responses were compared on the basis of participants’ SUS ratings. This allowed us to gain better insight into the relationship between identified themes from interviews and experienced usability.

## Results

### Interview Results

Saturation was reached after 13 interviews. Interviews lasted for approximately 20 to 50 minutes and had a mean duration of 33 minutes. Participants’ mean age was 42 years. A majority of participants were female (10/13, 77%). Further, 5 participants had an SUS score of <70, 5 participants had a score of between 70 and 80, and 3 participants had a score of >80. The demographic characteristics of the study population can be found in Table 1.

**Table 1:** Demographic characteristics.

Participant number	Age (years)	Sex	Educational level	Previous experience in blended care	Interest in technology in the field of health care	System Usability Scale score
1	35	Female	Intermediate	No	Yes	67.5
2	48	Female	Intermediate	No	No	60.0
3	38	Female	Intermediate	No	Neutral	77.5
4	23	Female	Intermediate	No	Neutral	57.5
5	42	Female	Basic	No	Neutral	55.0
6	42	Male	Intermediate	No	Yes	50.0
7	48	Female	High	Yes	Yes	77.5
8	43	Female	Intermediate	No	Yes	85.0
9	47	Female	High	Yes	Yes	80.0
10	38	Male	High	No	No	72.5
11	31	Female	High	No	Yes	72.5
12	52	Male	High	No	Yes	87.5
13	57	Female	High	No	No	95.0

The 13 interviewees formed a subset of participants from the PARASOL intervention arm (n=80; age: mean 47 years; female: 57/80, 71%). The overall averages of the PARASOL intervention participants were hence comparable to those who were selected for interviews on the basis of purposeful sampling. The overall mean SUS score in the PARASOL intervention arm (n=55) was 74.6. A total of 19 participants had an SUS score of <70, 15 participants had an SUS score of between 70 and 80, and 21 participants had an SUS score of >80. Further, 20 participants in the PARASOL intervention did not complete the intervention, and 5 questionnaires were not submitted.

As the use of e-coaching integrated in treatment is relatively new, participants were asked about their general experience with and interest in technology in health care. Every participant had used some form of technology (in the broadest sense of the word). The use of a PC, smartphone, and tablet were mentioned. The integration of technology in health care was only previously experienced by 2 of the participants. When asked about technology in health care, participants mentioned the use of pedometers, health apps, and websites. Participants’ interest in technology differed, as can be seen in Table 1.

A total of 4 themes emerged from the interviews. These themes provided insight into the usability of a blended approach to an integrated intervention from patients‘ perspectives.

### Theme 1: Motivations and Expectations Prior to Participation in the Intervention

There was no consensus on participants’ expectations prior to the intervention. Some participants stated that they had no expectations or that they had no expectations that their complaints would disappear by participating in the intervention. Others expected fewer complaints and more physical activity, and some expected that their pain would go away. A recurring statement reflected the hope that someone would seriously consider their complaints:

That someone finally thinks about the fact that these complaints are really there, and that a program is being made.

Participant #3

In terms of motivation, some participants participated mainly for personal interest. Other participants were just curious and saw no disadvantages, and some started the intervention because of a referral from their general practitioner. Experiencing intense pain was a motivation for participating in the intervention, and some participants mentioned that there were no other options for treatment with regard to their complaints. One participant stated:

I take this, because elsewhere a program is never really offered.

Participant #8

When the results were analyzed based on SUS score groups, they showed that higher overall SUS scores were related to quotes regarding autonomy and intrinsic motivation (Textbox 2). In terms of expectations related to the intervention program, there was no difference among SUS groups (Textbox 3).

**Quotes related to motivation. The quotes are stratified by System Usability Scale (SUS) score groups.**

**SUS score group: <70**

- “I participate to stay active”
- “Advice from GP”

**SUS score group: 70-80**

- “Interesting to see whether the mental and physical aspects come together”
- “I don’t understand my complaints and want to know what they are, and how I can deal with them”

**SUS score group: >80**

- “I have to make use of this opportunity, as I have been looking for ways to deal with my complaints for two years”
- “I had no way to resolve my complaints, and perhaps this will help me”

**Quotes related to expectations. The quotes are stratified by System Usability Scale (SUS) score groups.**

**SUS score group: <70**

- “I don’t know if it will work”
- “I have no idea what to expect”

**SUS score group: 70-80**

- “I’m curious, rather than have any expectations”
- “I thought, this must really work”

**SUS score group: >80**

- “I was open to something new”

### Theme 2: Applicability of e-Coaching

References were made to e-coaching during interviews twice. The first reference concerned the look and feel of the application, and the second concerned the application’s acceptability. Some participants mentioned that they spent a long time searching within the application and found the web-based portion to be confusing. For example:

I had to watch instruction videos but I could not find them.

Participant #3

Other participants however found the site to be well structured. There was no consensus on the ease with which documents or instruction videos could be found. Many participants had problems with logging in. In addition, the application often had bugs. This did not promote the use of e-coaching. One participant said:

I did my exercises every day but the program did not work so I just did not fill it in.

Participant #13

Another participant missed an evaluation that would have given insight into their progress. The ability to ask questions on web-based platforms and the fact that people can use the intervention anywhere were mentioned as facilitators. Participants stated that the planning assignments and exercises were clear every week. One participant said:

What I found very clear was that you could just click and do your exercises and activities on a weekly and daily basis.

Participant #8

Participants appreciated the ability to tick off the followed modules so that it was immediately clear which modules had been completed and which were still open. There was no consensus on whether obtaining information through text or film was preferred. Participants gave the following tips for the use of e-coaching:

Add forms on the site to leave notes on progress, e.g. how many minutes one walked.

Participant #9 and Participant #11

Make assignments more accessible by using visual support (colors, shapes).

Participant #7

The higher the satisfaction (as measured by the SUS), the more participants understood and used the web-based environment (Textbox 4).

**Quotes related to the applicability of e-coaching. The quotes are stratified by System Usability Scale (SUS) score groups.**

**SUS score group: <70**

- “It is unclear for me how to use the website”
- “I can’t enter the system, I never accessed the online part”
- “I often did not fill out the online sections, I prefer face-to-face treatments”

**SUS score group: 70-80**

- “I could not find the video, so I used text”
- “Clear and easy to use”

**SUS score group: >80**

- “The videos are clear and easy to use in daily life”
- “The site was clear”
- “It was easy to get the hang of the application”

### Theme 3: The Role of Health Care Professionals

An often-mentioned facilitator of the treatment was agreement among health care professionals. Participants felt that they were receiving the same information from different angles. In face-to-face treatments, which participants felt to be useful, health care professionals provided psychoeducation, in which reminders and repetition were introduced to patients. A participant stated:

Because both the mental health nurse and the physical therapist spoke about interpreting pain, for example, and the physical therapist explains it more anatomically.

Participant #11

The important roles of health care professionals were found to be discussing exercises, providing information, setting goals, and helping patients reach these goals. Participants also appreciated the fact that health care professionals supported reflections on behaviors and thoughts via confrontation, convincement, and motivation. For example:

Holding up a mirror to me, that there was a confrontation, it was very helpful that the physical therapist was confrontational.

Participant #12

Another facilitator was the approachability of the mental health nurse. Participants recommended increasing the involvement of the general practitioner to increase the amount of feedback and encouragement that they receive. One participant stated:

I can imagine that people with these complaints do not always immediately think the mental health nurse and the physical therapist are going to solve the problem, so I think that the GP is still important for encouragement.

Participant #3

Participants also did not expect physical therapists to engage in conversations as much as they did:

I think physical therapy is important only when giving exercises and not for conversations.

Participant #10

The higher the SUS score, the more patients understood that health care professionals acted as coaches rather than as therapists (Textbox 5). There was no difference among subgroups with regard to interprofessional collaboration (Textbox 6).

**Quotes related to the role of professionals. The quotes are stratified by System Usability Scale (SUS) score groups.**

**SUS score group: <70**

- “I feel the need to have my own say more”
- “Sometimes I feel I have the same conversation twice, the physical therapist and I were a better match and we could converse more easily”

**SUS score group: 70-80**

- “The physical therapist remember me and my story, and that made me feel good”
- “I expected more from the physical therapist, just conversing and no exercises”

**SUS score group: >80**

- “The professionals were very involved”
- “It’s good that the professionals held up a mirror to me”

**Quotes related to interprofessional collaboration. The quotes are stratified by System Usability Scale (SUS) score groups.**

**SUS score group: <70**

- “Good cooperation, same advice”
- “The same advices, did not notice cooperation, I did know they coordinated amongst the two of them”

**SUS score group: 70-80**

- “The combination of the mental health nurse and the physical therapist was good”
- “There was an overlap, but that did not bother me, it was complementary”

**SUS score group: >80**

- “I know they coordinated, they did not enter each other’s domains”
- “One was more physical, the other was more psychological”

### Theme 4: Integrated Design of the Blended Approach

Given that only 2 participants had previous experience with blended care, interview questions about this new method of delivering health care were asked. Some participants were satisfied with the higher frequency of face-to-face appointments at the start of the intervention, while others were not. The time between appointments increases the chance of forgetting parts of the treatments. The face-to-face sessions served as a reminder:

Because I forget a lot, so it's nice that I can have feedback reminder.

Participant #5

Participants suggested making the number of face-to-face sessions dependent on individual preferences. One participant said:

I think you should personally consult with each individual on the number of appointments.

Participant #13

Others indicated that the number of face-to-face appointments should be made dependent on one’s experience with web-based applications. For example:

I think for me personally I could have done with fewer appointments, as I am used to work online.

Participant #8

Participants also mentioned that it was important for face-to face sessions and e-coaching to be coordinated. One participant stated:

You are encouraged to do the online program and then you come to practice and can get the information again, it connects.

Participant #7

Another stated that face-to-face sessions filled the gap that was left on web-based platforms:

In fact, I first had to read the explanation on the website and then my questions were discussed.

Participant #10

The possibility to schedule therapy based on personal preferences however was seen as an advantage. For example:

I liked the times. It was possible for me to make an appointment at the end of the day.

Participant #7

The advantage of e-coaching was that participants could prepare specific questions that could be asked during the face-to-face sessions (eg, “I could ask specific questions I prepared myself” [Participant #10]). Further, participants generally perceived blended care as positive (eg, “But that you can check it yourself at home. I think this is very good” [Participant #7]).

Participants appreciated the integrated design of the intervention across all of the different SUS score groups (Textbox 7).

**Quotes related to the integrated design of the blended approach. The quotes are stratified by System Usability Scale (SUS) score groups.**

**SUS score group: < 70**

- “Because feedback is more specific for my own situation”
- “Face-to-face was a reminder...I find personal contact to be very important”

**SUS score group: 70-80**

- “The proportion [face-to-face and online] and frequency was good”
- “Face-to-face and online matched”
- “Repetition made it easier to remember”

**SUS score group: >80**

- “I find it easy to combine with other activities, I could do with less appointments”
- “The number of appointments should be based on personal preferences”

Overall, the results of this study show that participants experienced the intervention positively. This integrated blended care intervention aimed to promote self-management among patients and provide patients with insights into dealing with their complaints. Participants stated that they learned about self-management:

Now, I can estimate what I can do and cannot do.

Participant #9

I can actually do it all by myself.

Participant #8

Participants also gained more insights into dealing with their complaints:

Knowing nothing is broken, that idea has reassured me.

Participant #4

Because of graded activity, pain turns into pride; I am happier, undertake more, sing more; I'm enjoying more.

Participant #11

Textbox 8 includes all of the core themes that emerged from the semistructured interviews and hence summarizes usability from patients’ perspectives. It shows the factors that were appreciated and lessons learned for improving usability.

**Summary of findings.**

**Factors that patients appreciated**

- Information being recognizable
- The intervention as an incentive
- The personal approach
- The holistic approach
- Interprofessional collaboration

**Lessons learned for improving usability**

- Connect the intervention to the individual’s situation and motivation
- Improve the accessibility of and technology support in e-coaching
- Introduce the possibility of asking questions on web-based platforms
- Personalize the intervention with respect to the amount of personal guidance alongside e-coaching

## Discussion

### Principal Findings

In this study, we evaluated patients’ perspectives on the usability of an integrated blended care intervention. All included patients participated in a 12-week proactive blended care intervention in primary care with the aim of preventing the chronicity of MUPS. Participants were all generally positive about the received care. Various aspects of usability were highlighted, and responses were categorized into 4 themes.

The first theme that arose from interviews was the motivations and expectations of patients prior to the intervention. Existing literature shows that interventions that match patients’ expectations are more effective in achieving sustainable changes in patients [23]. This especially holds true for intrinsic motivation rather than extrinsic motivation, which increases one’s willingness to spend more time on assignments [34] and results in better health care outcomes [35]. Motivation also seems to be a factor of patients’ adherence to eHealth [36]. In this study, we found differences in motivation related to satisfaction. When the overall results of the interviews were compared based on SUS scores, intrinsic motivation seemed to be an important factor related to experienced usability. Another factor that may influence a patient's motivation is patient selection. In this study, an electronic screening method involving the use of data from the electronic medical records of general practitioners’ patients was used [29]. All eligible patients who were at risk for the chronicity of complaints were proactively approached by their general practitioners via an invitation letter. The selection of patients via this approach also has implications for patients’ motivation, as the chance of approaching patients who may be less motivated may increase. To achieve adherence in patients, one should therefore take motivation into account in future interventions.

Many participants were not satisfied with the technical support provided in e-coaching, as technical functions did not work and logging in was a problem. The degree of satisfaction, which was measured with the SUS, increases when the web-based environment is understood and can be used. When patients were uncertain about the usefulness of e-coaching, the e-coaching modules were not used. This phenomenon has also been found in literature. Adapting eHealth to users’ understanding and capabilities leads to a more usable and useful system [23]. When comparing the ages and educational levels of the participants in the low and high SUS score groups, a finding that stood out was that those with lower satisfaction were substantially younger and had lower educational levels. Existing literature shows that individuals with less education have worse actual and self-rated skills for evaluating the quality of web-based health information and lower trust in web-based health information compared to those with more education [37]. Studies however have found no consensus regarding the relationship between satisfaction and age [37].

Irrespective of the differences in satisfaction with e-coaching, participants were satisfied with the interprofessional collaboration. The holistic approach, through which physical therapists and mental health nurses provided information from different angles, was positively received by the participants. The expectations of participants regarding the role of health care professionals however differed among the SUS score groups. The higher the SUS score, the more patients understood that health care professionals acted as coaches rather than as therapists. Participants in the lower SUS score group, for instance, felt that they had to explain their complaints twice and expected that the roles of physical therapists would include more than just engaging in conversations and providing exercises. As the organization of health care has changed (ie, focusing more on prevention) [38], the role of health care professionals will also change; health care professionals will shift their focus from being a therapist to being more of a coach [39]. It seems important to explain this new role at the start of integrated blended care interventions in order to better shape the expectations of patients. Aside from interprofessional collaboration, attention should also be given to the collaboration between professionals and patients. Shared decision-making can support this process [40].

Participants appreciated the integrated design of the intervention across all of the different SUS score groups. They positively evaluated the possibility of saving texts and videos for future reference and the repetition of information in e-coaching combined with face-to-face sessions. The ability to personalize face-to-face sessions by allowing patients to prepare specific questions after studying the general information in the e-coaching modules was appreciated. Earlier studies have underlined the importance of face-to-face treatment combined with web-based care, as this has been found to improve and preserve outcomes [35,36,41]. The extent to which the intervention was tailored to participants made interventions and information recognizable. Participants also mentioned that an important yet missing part of the intervention was a diary or a free space for taking notes on exercises. The option to tick off exercises and modules and the explanation of exercises were considered to be helpful. These findings are supported by literature stating that the key components of the positive effect that eHealth has on health outcomes are personalization, stimulation, goal setting, and the integration of e-coaching [21]. All of these elements were available in the integrated blended care intervention.

The results of this study demonstrate the usability of an integrated blended care program for patients with MUPS. More research is needed to investigate whether these results are patient specific or whether the results of this patient population are unique. What remains important is ensuring that the use of technology in treatment fits the participants [42]. A checklist can help health care professionals, together with patients, to decide whether a patient is eligible for this program and whether the program matches a patient’s characteristics (eg, abilities, needs, and preferences) and prior experiences with blended care [41].

### Strengths and Limitations

A limitation of this qualitative study is that all information is based on a specific integrated blended care intervention—the PARASOL intervention. Therefore, some items of the core themes are directly linked to this specific intervention. However, recommendations are insightful in general when starting an integrated intervention with a blended approach.

The theoretical construct of Bleser et al [26] was chosen. This construct contains the performance, satisfaction, and acceptability features. Other theoretical constructs for gathering insights into usability also exist, such as the Unified Theory of Acceptance and Use of Technology and the Technology Acceptance Model. These other constructs however largely overlap [43,44]. The Unified Theory of Acceptance and Use of Technology focuses more on social influences related to behavioral intention, whereas the Technology Acceptance Model focuses on perceived usefulness and ease of use. Given the findings of this study, including other measuring instruments, such as the Intrinsic Motivation Inventory and the Rotter locus of control scale, could be an interesting addition in future research. These could shed more light on patients’ motivations at the start of the program. The strengths of this study are the use of an iterative process during the analysis of the results and the use of triangulation methods during the whole research process. Furthermore, patient involvement was sought in all research phases.

### Conclusions

The successful implementation of integrated blended care interventions based on patients’ perspectives requires matching treatments to patients’ individual situations and motivations. In addition, personalizing the relative frequency of face-to-face appointments and e-coaching is of importance.

## Abbreviations

MUPS: medically unexplained physical symptoms
SUS: System Usability Scale
